# Let’s do it right: Eight steps to competence in laboratory animal science in the European Union

**DOI:** 10.1177/00236772231157455

**Published:** 2023-03-24

**Authors:** Rafael Frías

**Affiliations:** Unit for Education and Training, Comparative Medicine, Karolinska Institute, Sweden

**Keywords:** Animal use, ETHICS & WELFARE, ETHICS & WELFARE, POLICY, Teaching & training, Teaching & training

## Abstract

Demonstrated competence in laboratory animal science (LAS) is a prerequisite in Directive 2010/63/EU to work with animals used in scientific procedures, as it is essential to increase animal welfare, improve the quality of science, promote the acceptability of animal research and meet the demands of free movement of personnel and scientific exchange. Although since 2010 there have been eight clear steps to achieving the required competence of personnel working with animals used in science, it is not uncommon to see documentation for individuals who have just completed an LAS course that contains only education and training elements (three steps), for which the status of competence in LAS is granted. Here, a simplified summary of how competence in LAS should be delivered in eight steps according to EU recommendations is presented.

## Bien faire les choses : 8 étapes vers la compétence en science animale de laboratoire au sein de l'Union européenne Résumé

La compétence démontrée en science des animaux de laboratoire (LAS) est une condition préalable de la directive 2010/63/UE pour travailler avec les animaux utilisés dans les procédures scientifiques, car il est essentiel d'accroître le bien-être des animaux, d'améliorer la qualité de la science, de promouvoir l'acceptabilité de la recherche animale, et de répondre aux exigences de la libre circulation du personnel et des échanges scientifiques. Bien qu’il existe depuis 2010 huit étapes claires pour atteindre la compétence requise du personnel travaillant avec les animaux utilisés dans la science, il n'est pas rare que la documentation destinée aux personnes qui viennent de compléter un cours de SAL ne contienne que des éléments d'éducation et de formation (trois étapes), pour lesquels le statut de compétence en SAL est accordé. Nous présentons ici un résumé simplifié de la manière dont la compétence en SAL doit être fournie en huit étapes, conformément aux recommandations de l'UE.

## 8 Schritte zur Kompetenz in der Versuchstierkunde in der Europäischen Union Abstract

Der Nachweis der Kompetenz in der Versuchstierkunde (LAS) gilt laut Richtlinie 2010/63/EU als eine Voraussetzung für die Arbeit mit Tieren, die in wissenschaftlichen Verfahren verwendet werden, da er wesentlich ist, um den Tierschutz zu verbessern, die Qualität der Wissenschaft zu erhöhen, die Akzeptanz der Tierforschung zu fördern und die Anforderungen der Personalfreizügigkeit und des wissenschaftlichen Austauschs zu erfüllen. Obwohl seit 2010 acht klare Schritte zur Erlangung der erforderlichen Kompetenz für Personal existieren, das mit in der Forschung verwendeten Tieren arbeitet, ist aus Unterlagen von Personen, die LAS-Kurse absolviert haben, zu ersehen, dass diese häufig nur Aus- und Weiterbildungselemente (drei Schritte) enthalten, für die der LAS-Kompetenz-Status gewährt wird. Im Folgenden wird eine vereinfachte Zusammenfassung darüber gegeben, wie die LAS-Kompetenz gemäß den EU-Empfehlungen in acht Schritten korrekt vermittelt werden sollte.

## Hagamos lo correcto: ocho pasos hacia la competencia en la ciencia de los animales de laboratorio en la Unión Europea Resumen

La competencia demostrada en la ciencia de los animales de laboratorio (LAS) es un requisito previo en la Directiva 2010/63/UE para trabajar con animales utilizados para experimentos científicos, ya que es esencial para aumentar el bienestar de los animales, mejorar la calidad de la ciencia, promover la aceptabilidad de la investigación con animales y satisfacer las demandas de la libre circulación de personal y el intercambio científico. Aunque desde 2010 existen ocho pasos bien claros para conseguir la competencia requerida del personal que trabaja con animales utilizados científicamente, no es raro ver documentación para personas que acaban de completar un curso de LAS que solo contiene elementos de educación y formación (tres pasos), mediante el cual se concede el estatus de competencia en LAS. A continuación, presentamos un resumen simplificado de cómo debe impartirse la competencia en LAS en ocho pasos según las recomendaciones de la UE.

Competence in healthcare professionals has been defined as the ability to perform roles and tasks to the expected standards.^1^ For a physician, this would in practice involve the acquisition and integration of high levels of knowledge, skills and attitudes.^2^ Laboratory animal science (LAS) is a multidisciplinary field of science that deals with scientific, societal, ethical, legal and optimal standards for the care and use of animals that will be used for scientific purposes as models for humans and other species. In LAS, a competent person would be one who is able to perform specific tasks and roles involving the care or use of animals for scientific or educational purposes to the expected standards and who demonstrates high levels of knowledge, skills and professional behaviours. Table 1 provides a summary of the key requirements for competence of personnel specified in the Directive. The roles and tasks for staff consist of carrying out procedures (function (a)), designing procedures and projects (function (b)), taking care of animals (function (c)) and killing animals (function (d)). The objective is to carry out such tasks in accordance with the three Rs and to the highest ethical, legal, scientific and veterinary standards.^3–5^

**Table 1.** Summary of key requirements for the competence of personnel specified in Directive 2010/63/EU.

Article	Description
Art. 23	(…) have species-specific knowledge.
	(…) be adequately educated and trained.
	(…) be supervised in the performance of their tasks until the requisite competence is demonstrated.
	(…) obtain and demonstrate the requisite competence.
	(…) maintain the requisite competence.
Art. 24	(…) be continuously trained.

**Figure 1.**
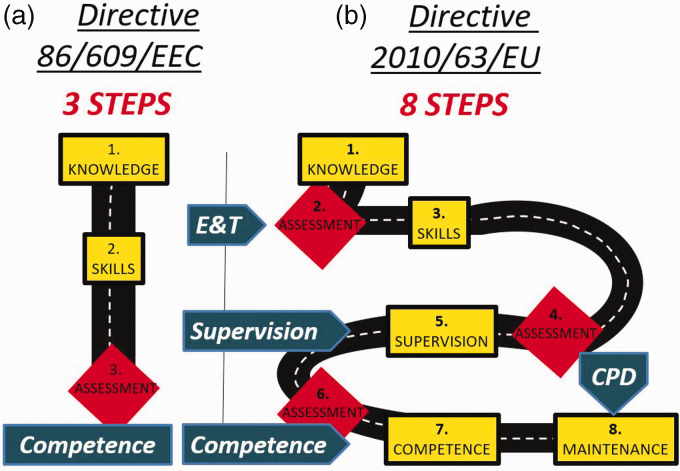
Difference between (a) three and (b) eight steps to competence in laboratory animal science. E&T: Education and Training; CPD: continuing professional development.

Between 1986 and 2010, completing a course in LAS to demonstrate competence was the traditional approach to be able to work with laboratory animals. During this period, a trainee was considered competent, for example, to carry out procedures on animals after completing an LAS course that included only education and training elements (three steps) (Figure 1(a)). This three-step approach was sufficient for a person to be considered legally competent to perform roles and tasks in LAS after attending a course lasting approximately 40 h that would include theoretical contents (step 1), practical sessions (step 2) and a final assessment at the end of the course (step 3).^6–8^ However, this three-step process to achieve competence in LAS falls short in meeting the current eight legal requirements (Figure 1(b)).^5^

Although since 2010 there have been clear and additional expectations specifically regarding the competence of personnel working with animals used in science,^5^ the traditional three-step approach still appears to be used in some institutions to deliver competence. In 2014, the Directorate-General for Environment at the European Commission published specific guidelines to fulfil the legal requirements for the competence of personnel.^9^ These guidelines have been endorsed by the national competent authorities of all Member States, and well-defined and effective statements such as competence not being delivered via education and training alone are presented throughout the text.

The process of achieving and maintaining the required competence in LAS in eight steps is summarized below.

## Step 1 – Education

Staff must have adequate education to acquire species-specific knowledge for functions (a) to (d). It is generally accepted that knowledge in LAS is acquired through the completion of educational modules after achieving specific learning outcomes. Learning outcomes are statements of what a trainee is expected to know, understand and be able to do at the end of a learning period. Outcomes are usually expressed as knowledge, skills or attitudes. Initial education is expected to deliver basic knowledge and understanding only.

## Step 2 – Assessment of knowledge

All the intended learning outcomes for each module should be mapped and assessed (‘blueprinting’). Knowledge must be adequate, and it should be assessed using methods that are valid (measuring what they are supposed to measure), reliable (providing stable and consistent results) and feasible (the method can be used). Examples of valuable methods for assessing knowledge include multiple-choice questions, extended matching questions, short-answer questions and pick-list questions.

## Step 3 – Training

Staff must receive adequate training to acquire skills for functions (a), (c) and (d). Practical training should be delivered by qualified trainers and should differentiate between minor procedures without anaesthesia (learning outcomes in EU modules 3.2, 6.2, 8) and major procedures with anaesthesia, analgesia and/or surgical techniques (learning outcomes in EU modules 20–22), depending on the eventual trainee’s role and the tasks to be performed on animals. An important consideration is the use of live animals for training purposes, which should strictly comply with the three Rs. Thus, the use of animals must be limited to when it is justified, the number of animals should be limited to the minimum and the severity of procedures should be limited to non-recovery or mild. Only those individuals who are at a stage in their career development where animal use is considered necessary should be offered the possibility to use animals for training purposes.

## Step 4 – Assessment *for* learned skills

Assessment is an action to obtain information from a candidate about their performance or competence. Assessment *for* learned skills is based on giving feedback to the trainee to help them improve their skills during a course, and assessment *of* learned skills is based on certifying and recording the trainee’s achievement at the end of the supervision period. Learning outcomes for practical skills during a course should be assessed to assign a level of supervision and to ensure that the trainee can proceed to working under supervision in a real working environment with no increased risk to animal welfare. The acquisition of adequate skills should be assessed by a qualified assessor using methods that are valid, reliable and feasible. Examples of methods of assessment *for* skills include direct observation of practical skills (DOPS) and objective structured clinical examination (OSCE). Assessment *for* skills in a formative environment can have significant benefits, such as increased trainee engagement, a more personalized learning experience, allowing the development of greater skills, and the promotion of reflective thinking. The skillset (knowledge, skills and attitudes) must be matched with the level of supervision. The higher the skillset, the lower the level of supervision that would be required.

## Step 5 – Working under supervision until the required competence is demonstrated

After initial practical training, working under supervision is most likely necessary to enable the development of a deeper understanding of the knowledge base, as well as proficiency in skills for functions (a), (c) and (d). The duration of the supervision period and time taken until competence is achieved will vary among trainees. Training under supervision allows for the learned skill sets to be applied in the local environment. Trainees with only cognitive skills (‘knowledge’ or ‘know-how’ to carry out a procedure)^2^ should work under ‘hands-on’ supervision, whereas trainees who are able to ‘show’ or ‘show how’ to do a procedure (but are unable to do it independently) should continue under ‘hands-off’ supervision.^9^ Only individuals who have been assessed as competent should work without supervision.

## Step 6 – Assessment *of* competence

Only individuals who have been assessed as competent can work without direct or indirect supervision. At this point, deeper understanding of the knowledge base, as well as proficiency in skills for functions (a), (c) and (d) should have developed. The acquisition of adequate skills can be assessed as in Step 4, but it should preferably take place in the trainee’s normal working environment. Other examples of assessment methods may include the mini-clinical evaluation exercise, mini-peer assessment tool, multi-source feedback, portfolios and workplace-based assessment. Assessment *of* competence in a summative environment can establish the level of progress that a trainee has made and will set the basis for competence certification where no supervision is required to work with animals.

## Step 7 – Demonstration of competence

Competence can decline when it is not properly maintained. This is the main reason why competence should be subject to continued review and quality assurance oversight is essential. A system should be in place at the establishment to ensure that poor practice in any member of staff is recognized and reported to allow appropriate corrective actions to be taken. Examples of quality assurance overseeing include periodic reviews of competence, random spot-checks, targeted checks following internal audits or after the results of retrospective reviews, or outcomes from incident reports.

## Step 8 – Maintenance of competence

Competence for all functions (a)–(d) should be considered as a continuing process that can be achieved, expanded and maintained through continuing professional development (CPD). CPD is the systematic maintenance, improvement and broadening of knowledge, skills and personal qualities necessary for the execution of professional and technical duties throughout the individual’s working life. It is important that most of the CPD activities are directly relevant to LAS. The Federation of European Laboratory Animal Science Associations (FELASA) has proposed guidelines for continuing education in LAS based on the awarding of credits or the number of hours of education.^10^

In conclusion, competence in LAS is essential for the implementation of optimal standards for the care and use of animals in science in our duty to ensure the highest possible health and welfare status of animals, to deliver scientific quality from in vivo models and to increase public trust and the acceptability of animal research. Here, a simplified version of the eight steps required to achieve and maintain this competence is provided to keep re-assuring humane and responsible care and use of animals for scientific purposes.

## Data Availability

The data availability statement does not apply to this article.

## References

[1] Eraut M. Concepts of competence. J Interprof Care 1998; 12: 127–139.

[2] Miller GE. The assessment of clinical skills/competence/performance. Acad Med 1990; 65: 63–67.2302301

[3] Dreyfus SE. The five-stage model of adult skill acquisition. Bull Science Technol Soc 2004; 24: 177.

[4] Pastor Campos A de la Cueva Bueno E Martín Zúñiga J , et al. SECAL Working Group Report – Guidelines for persons working under supervision in laboratory animal facilities. Journal of the Spanish Society of Laboratory Animal Science Association (JSECAL) 2018. https://secal.es/wp-content/uploads/2018/07/Guia-para-la-gestion-del-trabajo-bajo-supervision.pdf

[5] European Directive. Directive 2010/63/EU on the protection of animals used for scientific purposes. OJ L 276, 20.10.2010. 2010, pp.33–79.

[6] Council Directive 86/609/EEC of 24 November 1986 on the approximation of laws, regulations and administrative provisions of the Member States regarding the protection of animals used for experimental and other scientific purposes. OJ L 358 18.12, p.1, CELEX: https://eur-lex.europa.eu/legal-content/EN/TXT/?uri=CELEX:31986L0609 (1986, accessed 16 March 2023).

[7] FELASA. FELASA recommendations on the education and training of persons working with laboratory animals: Categories A and C. Reports of the Federation of European Laboratory Animal Science Associations Working Group on Education accepted by the FELASA Board of Management. Lab Anim 1995; 29: 121–131. 7602998 10.1258/002367795780740177

[8] FELASA. FELASA recommendations for the education and training of persons carrying out animal experiments (Category B). Report of the Federation of European Laboratory Animal Science Associations Working Group on Education of Persons Carrying out Animal Experiments (Category B) accepted by the FELASA Board of Management. Lab Anim 2000; 34: 229–235. 11037115 10.1258/002367700780384672

[9] European Commission, Directorate-General for Environment. Publications Office. Caring for animals aiming for better science: Directive 2010/63/EU on protection of animals used for scientific purposes: Education and training framework, https://data.europa.eu/doi/10.2779/311480 (2019, accessed 16 March 2023).

[10] Guidelines for continuing education for persons involved in animal experiments – Recommendations of a FELASA Working Group. Smith D, Dorier A, Hack R, Sjöquist M, Visa J, Zeller W, Ruane B, de Smet B, Weiss J. 2010 Jan. https://felasa.eu/working-groups/guidelines/id/20

